# A new era has begun: Treatment of atopic dermatitis with biologics 

**DOI:** 10.5414/ALX02259E

**Published:** 2021-08-27

**Authors:** Dora Stölzl, Stephan Weidinger, Katharina Drerup

**Affiliations:** Department of Dermatology, Venereology and Allergology, UKSH, Campus Kiel

**Keywords:** atopic dermatitis, biologics, systemic therapy, clinical trials

## Abstract

The era of biologics for the treatment of moderate-to-severe atopic dermatitis (AD) began in 2017 with the approval of dupilumab, a monoclonal antibody that binds to the α-subunit of the interleukin IL-4 receptor. Until then, only conventional immunosuppressants were available for systemic treatment, of which only cyclosporine is approved for the treatment of severe AD. In the meantime, the therapeutic landscape of AD has been changing rapidly, and additional biologics have been developed which target IL-13, the IL-31 receptor, OX40, and OX40L, among others. Many of these substances have already shown promising results in phase 1, 2, and in some cases also phase 3 trials. In June 2021, tralokinumab, an IL-13 antibody, has been approved in Europe for the treatment of moderate-to-severe AD in adults. In addition to antibody-based therapies, “small molecules” that, e.g., inhibit Janus kinases enrich the armamentarium of systemic AD therapies. With all these agents, not only will many more targeted therapies become available, but also will the complex and heterogeneous pathophysiological processes of this disease be better understood.

## Introduction 

**Figure 1.**
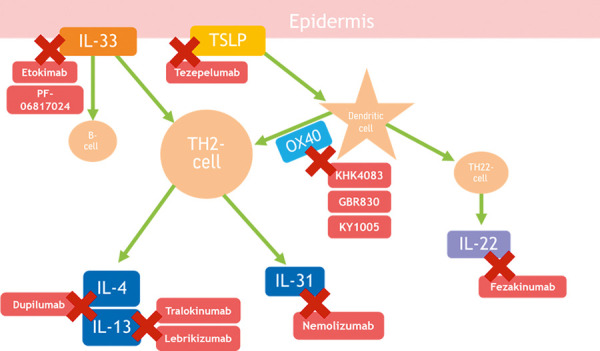
Biologics and their targets for atopic dermatitis treatment.

Atopic dermatitis (AD) is one of the most common chronic inflammatory skin diseases. For a long time, only conventional immunosuppressants were available to treat severe forms of AD that could not be adequately controlled with topical treatment alone [1]. With the approval of two “biologics” and the expected approval of further biologics as well as so-called “small molecules” that inhibit e.g. Janus kinases, the therapeutic landscape is now rapidly changing. This article presents an overview of biologics that are approved or are in development for the treatment of AD (Figure 1). 

## Dupilumab – the first biologic for the treatment of atopic dermatitis in adults and children (anti-IL-4Rα antibody) 

Dupilumab was the first biologic for the treatment of AD and has been available in Germany since 2017. It is approved for the treatment of moderate-to-severe AD in adults and adolescents aged 12 years and older who are eligible for systemic therapy, as well as for children aged 6 years and older with severe AD [2]. Phase 3 trials in children 6 months and older are ongoing. Approval studies from 6 months of age are still outstanding (NCT02612454, NCT03346434). Dupilumab is also approved for the systemic treatment of asthma with type 2 inflammation and severe chronic rhinosinusitis with nasal polyps. Dupilumab is a monoclonal IgG antibody that binds to the α-subunit of the interleukin (IL)-4 receptor and so, inhibiting the signaling pathways of the key type 2 cytokines IL-4 and IL-13 [3]. 

The efficacy and tolerability of dupilumab have been demonstrated in numerous randomized controlled trials. In two pivotal phase 3 monotherapy trials (SOLO1 and SOLO2), 671 patients with moderate-to-severe AD received dupilumab 300 mg or placebo s.c. every week (qw) or every other week (q2w) for 4 months. After 16 weeks, 37% of patients treated with dupilumab had achieved complete or almost complete symptom clearance compared to 9% in the placebo group. There was at least a 75% reduction of the Eczema Area and Severity Index (EASI) in 52% of dupilumab-treated study participants compared to only 15% of placebo-treated participants. In the LIBERTY AD CHRONOS phase 3 study, in which all patients were also allowed to use topical glucocorticosteroids (TCS) and/or topical calcineurin inhibitors (TCI), 39% of the dupilumab patients achieved complete or almost complete clearance of the skin after 16 weeks vs. 12% in the placebo group. A 75% reduction in EASI was achieved by 64% (dupilumab qw + TCS/TCI) and 69% (dupilumab q2w + TCS/TCI) vs. 23% (placebo + TCS/TCI). This improvement was maintained over the 52-week treatment period [4]. In 2018, the LIBERTY AD CAFE study investigated a patient population with an insufficient response, side effects or contraindications to therapy with ciclosporin. Dupilumab was also convincing here with the same effectiveness as in the other phase 3 studies [5]. In all trials, dupilumab was also more effective than placebo in other important dimensions of the disease, such as itching, sleep disturbance, and quality-of-life parameters [3, 4, 5, 6]. 

Recently, observations from “real-world studies” such as the German AD Registry TREATgermany largely confirmed the trial data [7, 8]. The safety profile of dupilumab is highly favorable, with local reactions at the injection site and eye symptoms (especially conjunctivitis) being the most common side effects, occurring in up to 28% of dupilumab-treated patients in clinical trials [9] and up to 38.2% in “real-world studies” [7]. Most cases of conjunctivitis appear to be mild and transient and do not lead to a discontinuation of treatment. Aszodi et al. [10] published a treatment algorithm for this adverse event which is easy to apply in practice. In severe cases, interdisciplinary treatment together with experienced ophthalmologists should be initiated. No increased risks of severe systemic infections or bacterial skin infections have been observed during treatment with dupilumab. Likewise, no clinically meaningful changes were observed for laboratory parameters, and thus laboratory tests are not required before initiation or during treatment. Recent 3-year data from an open-label extension study in adults at 300 mg weekly showed sustained efficacy with no additional safety signals; however, long-term and rare side effects have been poorly evaluated in the studies to date [11, 12]. 

The efficacy and safety of dupilumab in adolescents aged 12 – 18 years and children aged 6 years and older are comparable to that of adults. The dosage is adjusted to the body weight (BW) of the adolescent patients: If the BW is over 60 kg, the antibody is administered as in adult patients. If the BW is less than 60 kg, 400 mg dupilumab is administered as the initial dose, followed by 200 mg s.c. every 2 weeks [13]. 

A study in younger children (≥ 6 months to < 6 years) investigating the safety and efficacy of dupilumab after a single dose also showed a positive safety profile. The pediatric patients received either 3 mg/kg BW or 6 mg/kg BW. The EASI reduction was 44.6% in the 3 mg/kg BW group and 49.7% in the 6 mg/kg BW group after 3 weeks. An EASI75 was achieved by 30% and 20%, respectively. At week 4, all groups showed a worsening of the severity parameters, regardless of the dosing [14]. 

## Tralokinumab (anti-IL-13 antibody) 

Tralokinumab, an interleukin-13 antibody, was approved by the EMA in June 2021. 

Tralokinumab neutralizes the biological activity of IL-13, the key cytokine of type 2 inflammation in the skin, by inhibiting its binding to IL-13Rα1 and IL-13Rα2 [15]. In the two phase 3 monotherapy trials, ECZTRA1 and ECZTRA2, about twice as many patients achieved complete or near complete clearance of the skin (IGA score 0 or 1) after 16 weeks of tralokinumab treatment (15.8 and 22.3%, respectively) than with placebo (7.1% and 10.9%). Tralokinumab was also superior to placebo in terms of important secondary endpoints such as improvement of pruritus and quality of life [16]. In the phase 3 combination trial ECZTRA3 (concomitant class 3 topical glucocorticosteroid), 38.9% of tralokinumab-treated and 26.2% of placebo-treated patients achieved an IGA 0/1 at week 16. An EASI75 was achieved by 56.0% in the active-agent group compared to 35.7% in the placebo group [17]. In the initial 16-week treatment period, the frequency of adverse events was similar between tralokinumab and placebo in all three studies. Conjunctivitis, a side effect of particular interest because it is a common adverse event of dupilumab treatment, occurred more frequently with tralokinumab than with placebo in all studies (ECZTRA3: 11.1% vs. 3.2%). However, most cases were mild or moderate. Apart from injection site reactions, no clear signals for other clinically relevant side effects were observed [16, 17]. 

In all three trials, patients were re-randomized after week 16. In ECZTRA1 and 2, patients treated with tralokinumab who had achieved an IGA score of 0/1 or an EASI75 were allocated in a 2 : 2 : 1 ratio to tralokinumab 300 mg every 2 weeks or every 4 weeks or placebo for 36 weeks of maintenance treatment. Patients who had also achieved clinical response criteria with placebo continued to receive placebo. Patients who had not achieved clinical response criteria at week 16 were switched to open-label treatment with tralokinumab 300 mg every 2 weeks. In this second phase of the trial, 60% and 56% of patients with early response, who were continued on tralokinumab every 2 weeks also had an EASI75 at week 52, and interestingly, 49% and 51% of those who were switched to 4 weeks of treatment also had an EASI75. Unexpectedly, a proportion (33% and 21%) of patients who were switched to placebo at week 16 also maintained this response at week 52 [16]. 

In ECZTRA3, 92.5% and 90.8% of patients with an early response who were continued on tralokinumab every 2 and 4 weeks still had an EASI75 at week 32. 55.8% of the patients who had not yet shown an adequate response at week 16 achieved an EASI75 at week 32. These observations indicate that in some of the patients with an initially very good response, the dose can be reduced in the further course of treatment, whereas in some of the patients who do not show a sufficient response during the first 16 weeks, response may continue to develop with continued treatment [17]. Results from the ECZTRA6 monotherapy study in adolescents and the ECZTRA7 combination study in adults with inadequate response, side effects or contraindications to cyclosporine are expected soon. In addition, a monocentric investigator-initiated study investigating the effects of tralokinumab on skin barrier and irritability is still recruiting (NCT04556461). 

Tralokinumab was approved by the EMA on June 22, 2021 for the treatment of adults with moderate-to-severe AD. The Summary of Product Characteristics (SmPC) states that in patients who have achieved an symptom-free or near-symptom-free skin after 16 weeks of treatment, reduced dosing with application of 300 mg s.c. every 4 weeks may be considered at the discretion of the prescriber, whereas patients with an initial partial response may continue to benefit from treatment every 2 weeks beyond 16 weeks. If there is no response after 16 weeks of treatment, discontinuation should be considered [18]. 

## Other biologics 

In addition to the approved biologics dupilumab and tralokinumab, other biologics are currently in clinical trials. The most advanced are antibodies against IL-13 and the IL-31 receptor. In addition, IL-33, OX40, OX40L, and IL-1α are target structures of biologics in advanced phases of clinical trials (Table 1). 

### Lebrikizumab (anti-IL-13 antibody) 

Lebrikizumab is another monoclonal antibody against IL-13 that selectively prevents the formation of the IL-13Rα1/IL-4Rα heterodimer receptor signaling complex. Because its binding differs from that of tralokinumab, its efficacy and safety profile may differ. In a double-blind phase 2 study, adults with moderate-to-severe AD were randomized 1 : 1 : 1 to lebrikizumab 125 mg as a single dose, lebrikizumab 250 mg as a single dose, lebrikizumab 125 mg every 4 weeks for 12 weeks, or placebo every 4 weeks for 12 weeks. At week 12, significantly more patients who received lebrikizumab 125 mg every 4 weeks (82.4%) than placebo (62.3%) achieved the primary endpoint EASI50. An IGA 0/1 was achieved by 44.6% in the active-agent group and 15.3% in the placebo group (p < 0.001) [19]. Adverse events were similar between groups and were mostly mild or moderate. Interpretation of results is limited by the fact that topical glucocorticosteroids had to be applied twice daily 2 weeks before the start of administration as well as during the study. 

In another phase 2 trial with lebrikizumab as a monotherapy, adult patients with moderate-to-severe AD were randomized 2 : 3 : 3 : 3 to placebo every 2 weeks or to lebrikizumab 125 mg every 4 weeks after an initial “loading dose” of 250 mg at baseline and week 2, 250 mg every 4 weeks after a 500 mg loading dose at baseline and week 2, or 250 mg every 2 weeks after a 500 mg loading dose at baseline and week 2 (NCT03443024). The primary endpoint was the percent change in EASI from baseline to week 16. Compared with placebo (–41.1%), all lebrikizumab groups showed a dose-dependent statistically significant improvement: 125 mg every 4 weeks (–62.3%), 250 mg every 4 weeks (–69.2%), and 250 mg every 2 weeks (–72.1%). As a secondary endpoint, EASI75 was achieved by 24.3% of placebo-treated patients and 43.3%, 56.1%, and 60.6% of lebrikizumab-treated patients, respectively. Lebrikizumab was also shown to be superior to placebo concerning other important endpoints such as itch reduction and quality of life improvement at all doses studied. Treatment-related adverse events were reported only slightly more frequently in the lebrikizumab groups than in placebo and were mostly classified as mild-to-moderate in severity and did not lead to treatment discontinuation. Conjunctivitis was observed in 2.6% of lebrikizumab-treated patients vs. 0% in the placebo arm. In this study, TCS use was allowed as “rescue medication” [20]. The efficacy and safety of lebrikizumab is currently being evaluated in multiple phase 3 studies, including two 52-week studies, each with 16 weeks of induction treatment and 36 weeks of long-term maintenance therapy in patients 12 years of age and older (NCT04178967, NCT04146363). 

### Nemolizumab (anti–interleukin-31 receptor antibody) 

Nemolizumab targets the α-subunit of the IL-31 receptor and thereby inhibiting the pruritogenic, pro-inflammatory, and barrier-regulatory effects of IL-31 [21]. In a 12-week phase 2 study, 264 patients were randomized to receive placebo every 4 weeks (q4w) or nemolizumab s.c. at four different doses (0.1, 0.5, or 2.0 mg/kg BW q4w or 2.0 mg/kg BW q8w). At week 12, the primary endpoint of average pruritus was measured using a visual analogue scale (VAS). Here, patients achieved a –20.9% reduction in adjusted mean pruritus under placebo, and in contrast, a –43.7% reduction under 0.1 mg/kg nemolizumab (p = 0.002), a –59.8% reduction under 0.5 mg/kg (p < 0.0001), and a –63.1% reduction under 2.0 mg/kg q4w (p < 0.0001). The secondary endpoint observed was the EASI reduction. There was an EASI reduction of adjusted mean scores of –26.6 points in the placebo group, –23.0 points under 0.1 mg/kg nemolizumab, –42.3 under 0.5 mg/kg nemolizumab, and –40.9 under 2.0 mg/kg nemolizumab q4w. 11% of patients in the placebo group achieved an IGA improvement of at least 2 points at week 12 compared to baseline vs. 14%, 38%, and 24% in the nemolizumab groups (0.1 mg/kg, 0.5 mg/kg, 2 mg/kg q4w). Adverse events were similar between the nemolizumab groups and the placebo group. The most common were exacerbation of AD, nasopharyngitis, and upper respiratory tract infections [22]. 

In another phase 2 study, patients were treated with 10, 30 or 90 mg of nemolizumab or placebo q4w s.c. for 24 weeks. The use of topical steroids was allowed. Here, the 30-mg dosage was shown to be most effective. The EASI reduction compared to placebo was –68.8% vs. –52.1% (p = 0.028). At week 24, 36.8% of patients treated with 30 mg nemolizumab achieved an IGA 0/1 compared to 21.1% of patients in the placebo arm (p = 0.06). “Peak itch”/”peak pruritus” as measured by VAS was shown to be reduced by –67.3% in the 30-mg nemolizumab group at week 24 compared to –35.8% in the placebo group (p < 0.0001). The most common adverse events were nasopharyngitis and upper respiratory tract infections [23]. 

A post-hoc analysis of the phase 2b study on the subpopulation of patients with an EASI ≥ 16 only showed a significantly greater pruritus reduction at day 2 in the group treated with 30 mg nemolizumab (–22.8% vs. –12.3% reduction in peak itch as measured by VAS, p = 0.005). At week 16, the reduction was –68.5% vs. –30.9% in the placebo group (p < 0.001). An EASI75 was achieved at week 16 by 50.0% of patients in the active-agent group and 15.9% in the placebo group (p < 0.0001). IGA 0/1 was achieved by 32.0% vs. 6.8% (p = 0.002) [24]. 

A long-term-extension 64-week study was completed by 153 patients. Itch reduction from baseline to week 64 was highest in the group receiving 0.5 mg nemolizumab per kg BW (–89.6%); a –73.0% reduction was seen with 0.1 mg/kg nemolizumab, –74.7% with 2.0 mg/kg q4w, and –79.1% with 2.0 mg/kg q8w. The EASI reduction was –68.5%, –75.8%, –78.9%, and –69.3% in the 0.1 mg/kg, 0.5mg/kg, 2 mg/kg q4w, and 2 mg/kg q8w groups, respectively. No new adverse events were noted [25]. 

In a 16-week phase 3 study, Japanese patients with atopic dermatitis with moderate-to-severe pruritus and inadequate response to topical therapy were randomized 2 : 1 to receive either 60 mg nemolizumab every 4 weeks or placebo s.c.. Concomitant topical therapy was allowed. The primary endpoint was change in pruritus. The mean pruritis score at baseline was 75 (scale of 0 – 100, with higher scores indicating more pruritus). At week 16, the verum group showed a reduction of –42.8% compared to –21.4% in the placebo group (p < 0.001). The mean reduction in EASI in the nemolizumab group was –45.9% vs. –33.2% in the placebo group [26]. 

Currently, several phase 2 and 3 trials for adults and adolescents are ongoing (e.g., NCT03985943, NCT03989349, NCT03989206). 

### KHK4083 (anti-OX40 antibody) 

KHK4083 is a monoclonal antibody against OX40 (CD134). OX40 is a co-stimulatory receptor of the tumor necrosis factor (TNF) family expressed on activated CD4 T cells, memory CD4 cells, and T-regulatory cells, among others. It is activated by the ligand OX40L which is primarily expressed by antigen-presenting cells and contributes to the maintenance of T-cell responses [1]. In an open-label phase 1 study with 22 participants, patients showed a significant EASI improvement over 22 weeks with a good safety profile. An EASI50 was achieved by 65% and an EAS75 by 55.0% at week 22 [27]. An initial phase 2 study has now been completed, but results have not yet been published (NCT03703102). 

### GBR830 (anti-OX40 antibody) 

GBR830 is also a monoclonal antibody against OX40. In a phase 2a study, 76.9% in the verum group vs. 37.5% of patients in the placebo group achieved an EASI50 at day 71. The study’s limitation was that due to its low power it was not possible to detect a significant difference. The safety profile of GBR830 was comparable to placebo [28]. 

### KY1005 (anti-OX40L antibody) 

KY1005 targets the OX40 ligand. A phase 2a study was recently completed, but detailed results are not yet available. However, the manufacturer Kymab announced in a press release that KY1005 was superior to placebo for both primary endpoints (safety and EASI percentage reduction at week 16) [29]. 

### Tezepelumab (anti-thymic stromal lymphopoietin antibody) 

Tezepelumab is an antibody against the cytokine “thymic stromal lymphopoietin” (TSLP). In a phase 2a study, there was no significant difference in EASI50 compared to placebo at week 16 (64.7% vs. 48.2%, p = 0.091). Topical glucocorticosteroids were allowed [30]. 

### Etokimab (ANB020) (anti-IL-33 antibody) 

Etokimab targets IL-33 as a monoclonal antibody. As the primary endpoint after 16 weeks (a significant difference in EASI reduction compared to the placebo group) was not met in all etokimab doses, a phase 2b study was stopped early [31]. 

### PF-06817024 (anti-IL-33 antibody) 

Another IL-33 antibody is PF-06817024, which has been investigated in a phase 1 trial in healthy patients as well as in patients with moderate-to-severe AD. The study recruitment has been completed. No results are currently available (NCT02743871). 

### Fezakinumab (anti-IL-22 antibody) 

Fezakinumab is a monoclonal antibody against IL-22. In a phase 2 study, there was a higher rate of patients achieving SCORAD30 and SCORAD50 compared to the placebo group. However, this was not statistically significant. Larger and also significant differences in reduction in severity were seen in the patient population who had a SCORAD ≥ 50 at inclusion. An EASI was not collected in this study. Fezakinumab showed a favorable safety profile [32]. Currently, no phase 3 studies are ongoing. 

### Bermekimab (anti-IL-1 α antibody) 

Bermekimab targets IL-1α. In a proof-of-concept study, 71% of patients achieved an EASI75 after 7 weeks. However, this study was not placebo controlled [33]. Further (partly placebo-controlled) phase 2 trials investigating the safety and efficacy are being performed (NCT03496974, NCT04021862, NCT04791319). 

### Mepolizumab (anti-IL-5 antibody) 

Mepolizumab, an IL-5 antibody, did not achieve a significant difference in PGA, SCORAD and pruritus reduction compared to placebo in a phase 2 study of 40 patients (18 mepolizumab, 22 placebo) [34]. Another trial was stopped early after an interim analysis showed that predefined futility criteria were met (NCT03055195). 

## Outlook 

In addition to the approved biologics dupilumab and tralokinumab, many other biologics, primarily targeting mediators of type 2 inflammation, are in advanced phases of clinical development for the treatment of moderate-to-severe AD. Additionally to antibody-based therapies, “small molecules” that e.g. inhibit Janus kinases enrich the armamentarium of systemic AD therapies. All this together will further drive the change in the management approach in AD. Clinical and molecular observations under targeted therapy will also offer important new insights into the pathophysiology of this heterogeneous and complex disease. 

## Funding 

None. 

## Conflict of interest 

D. Stölzl – none; K. Drerup – none; S. Weidinger – For the following companies, there is cooperation within the context of clinical studies and/or lecturing and consulting activities: Sanofi Genzyme, Regeneron, LEO Pharma, AbbVie, Pfizer, Almirall, Galderma, GSK, Kymab. 

**Table 1.** Biologics for the treatment of atopic dermatitis in clinical trials.

Agent	Mechanism	Trial Phase
Lebrikizumab	Anti-IL-13 mAb	Phase 3
Nemolizumab	Anti-IL-31RA mAb	Phase 3
GBR830	Anti-OX40 mAb	Phase 2
KY1005	Anti-OX40 mAb	Phase 2
Tezepelumab	Anti-TSLP mAb	Phase 2
ANB020/Etokimab	Anti-IL-33 mAb	Phase 2, early terminated
PF-06817024	Anti-IL-33 mAb	Phase 1
Fezakinumab	Anti-IL-22 mAb	Phase 2
Bermekimab	Anti-IL-1α mAb	Phase 2
